# Improving Vitamin D Status and Related Health in Young Women: The Safe-D study – Part B

**DOI:** 10.2196/resprot.5465

**Published:** 2016-05-10

**Authors:** Marjan Tabesh, Suzanne Marie Garland, Alexandra Gorelik, Alison Nankervis, Skye Maclean, Emma Teresa Callegari, Shanton Chang, Kayla Heffernan, John Dennis Wark

**Affiliations:** ^1^ Department of Medicine Royal Melbourne Hospital University of Melbourne VIC Australia; ^2^ Murdoch Childrens Research Institute Royal Children’s Hospital VIC Australia; ^3^ Women’s Centre for Infectious Diseases Royal Women’s Hospital VIC Australia; ^4^ Department of Obstetrics and Gynaecology University of Melbourne VIC Australia; ^5^ Melbourne EpiCentre Royal Melbourne Hospital VIC Australia; ^6^ Diabetes Service Royal Women's Hospital VIC Australia; ^7^ Department of Computing and Information Systems University of Melbourne VIC Australia; ^8^ Bone and Mineral Medicine Royal Melbourne Hospital VIC Australia

**Keywords:** vitamin D, young women, health outcomes, intervention, Safe-D study, behavioral intervention, app, vitamin D supplementation, eHealth

## Abstract

**Background:**

Vitamin D deficiency is highly prevalent and associated with increased risk of a number of chronic health conditions including cardiovascular disease, poor bone and muscle health, poor mental health, infection, and diabetes. Vitamin D deficiency affects millions of Australians, potentially causing considerable suffering, economic loss, and mortality.

**Objective:**

To measure the effectiveness of a (1) mobile-based app (behavioral) and (2) pharmacological intervention to increase circulating 25-hydroxyvitamin D (serum 25 OHD) levels and health outcomes over 4 months of intervention compared with usual care in a cohort of young women with suboptimal serum 25 OHD levels (25-75 nmol/L).

**Methods:**

Participants with 25 OHD levels 25 to 75 nmol/L are invited to participate in this study. Participants are randomized to one of three groups in 1:1:1 ratio: a mobile phone–based application, vitamin D supplementation (1000 IU/day), and a control group. Data collection points are at baseline, 4, and 12 months post baseline with the major endpoints being at 4 months. A wide-range of information is collected from participants throughout the course of this study. General health, behavioral and demographic information, medications, smoking, alcohol and other substance use, health risk factors, nutrition, eating patterns and disorders, and mental health data are sourced from self-administered, Web-based surveys. Clinical data include anthropometric measurements, a silicone skin cast of the hand, cutaneous melanin density, bone mineral density, and body composition scans obtained through site visits. Main analyses will be conducted in two ways on an intention-to-treat (ITT) basis using the last observation carried forward approach as an imputation for missing data, and on a per protocol basis to compare the intervention arms against the control group at 4 and 12 months.

**Results:**

Publication of trial results is anticipated in 2017.

**Conclusions:**

The study will allow assessment of the effects of a mobile-based app behavioral intervention and vitamin D supplementation on vitamin D status and will evaluate the effects of improving vitamin D levels on several health outcomes.

## Introduction

### Background

Low vitamin D status is an issue of concern in today’s society and its prevalence has been reported to be high, with approximately 50% of the world population thought to be affected [1]. Low vitamin D levels have been shown to be associated with an increased risk of numerous chronic health conditions, including poor musculoskeletal health and cardiovascular disease [2]. Vitamin D deficiency also impacts on young women’s ability to achieve optimal peak bone mass increasing the risks of osteoporosis and osteoporotic fractures, which are major public health problems in the aging population [3,4]. Twenty-seven percent of women across Australia achieve optimal vitamin D levels (defined as serum 25-hydroxyvitamin D (25 OHD) greater than 75 nmol/L) [5]. Individual factors such as habitual sun exposure and skin color are likely to play major roles in determining vitamin D status [5,6]. Vitamin D deficiency is an important health risk factor for young women, particularly during their childbearing years, when deficiency can harm both the mother and the unborn child [7]. Despite the potentially serious effects of vitamin D insufficiency (serum 25 OHD between 26 and 49 nmol/L) and deficiency (serum 25 OHD <25 nmol/L), very few vitamin D studies have focused on young women; the majority of studies in the literature have studied general population cohorts or have exclusively focussed on elderly participants [8]. This study is of particular importance as many risks factor for certain cancers, autoimmune diseases, infections, neurological diseases, diabetes, and poor mental health are established during youth [1]. Vitamin D status is one of the factors that may impact on these disease risks.

The Safe-D study aims to (1) examine the links between vitamin D and various health indicators in a young female cohort (part A) [9], and (2) evaluate the effectiveness of a smartphone app compared with vitamin D supplementation in improving both serum 25 OHD levels and several health measures that have been associated with vitamin D deficiency (part B). By focusing on a younger cohort and using a successful recruitment strategy via social media [10], the study aims to achieve a much larger sample size of this population than previous research [11,12]. In addition, the relationship between vitamin D status and health will be comprehensively studied using state-of-the-art information technology-based data collection methods that have not been used in any similar studies, which have based results largely on self-reported data [13].

While vitamin D deficiency is associated with many poor health outcomes, its potential impact on young Australian women’s health has yet to be established. Moreover, there are evidence gaps about the safest and most effective interventions to improve vitamin D status. Of importance, much of the previous research has been limited by imprecise 25 OHD measurements [2].

Addressing factors that can lead to vitamin D deficiency earlier in life might be beneficial for long-term health, productivity, and quality-of-life of young women. Part A of the Safe-D study is described elsewhere [9]. Part B, a randomized controlled trial involving the use of a digital Web-based smartphone app, is described here.

### Study Rationale

This study focuses on 16- to 25-year-old women, because of: (1) the high prevalence of vitamin D deficiency in young people [1,2,14,15], (2) the importance of this life stage, as individuals become more autonomous and independent, and individual environmental as well as behavioral factors play an increasing role in shaping health patterns that have long-term consequences [16], (3) the popularity in this demographic of communication using mobile and social media technologies with which we have previous experience, and harness in this study [10], (4) vitamin D deficiency impacting young women’s bone mass increasing the risks of osteoporosis and osteoporotic fractures later in life [17], and (5) women’s smaller skeletons make them more likely to develop osteoporosis due to biological sex differences in the skeleton with ageing [18].

While there have been many studies examining the associations between vitamin D and health, only small, restricted studies have been conducted with young women. Therefore, this study aims to ensure that comprehensive health data are collated for this population subgroup.

## Methods

### Study Design

The Safe-D study comprises two distinct, but overlapping and interrelated components. Part A is a cross-sectional study of healthy women aged 16 to 25 years, aimed at investigating associations between 25 OHD levels and musculoskeletal health (bone density, bone turnover markers, muscle function), mood/mental health, body composition and weight, and atopic/allergic symptoms [9]. Part B is an open-label, blinded-endpoint, randomized controlled trial with three arms; a behavioral intervention, a pharmacological intervention, and control group [19,20]. Study participants are monitored for a period of 12 months. A comprehensive, Web-based questionnaire is completed by all participants at 0 and 12 months; an abbreviated version of this survey is completed at 4 months. The survey links are sent through Limesurvey (an open-source, password-protected, secure software survey tool) to each participants [21]. The questionnaires comprise five modules, which the participants are able to complete either altogether or on separate occasions over a 2-week period before their site visit. The modules cover health areas including demographics, medical history, use of health care professionals, use of medications and allergy data to establish current and/or past health conditions [14]. Nutrition, dietary behaviors, and weight management data are collected to identify change in body composition and weight and investigate the relationship between obesity and vitamin D. Body image, alcohol use, tobacco use, and illicit drug use information is collected because it has been suggested that vitamin D has a neuroprotective effect on dopaminergic pathways in the adult brain and may have a role in the management of drug dependence, and also smoking and alcohol use can affect vitamin D status [22]. Diet, physical activity, pain and injuries, sun exposure, and mental health data are collected to assess dietary intake of vitamin D, to control for physical activity as a contributor to weight and to investigate a relationship between vitamin D and musculoskeletal health. Sun exposure is collected to investigate a relationship between change in vitamin D and ultraviolet (UV) exposure.

Dietary intake is a confounder for the relationship between vitamin D and obesity [23]. The Cancer Council Victoria Questionnaire (comprehensive dietary questionnaire) is used to evaluate the diet type and portion sizes [23].

**Figure 1:**
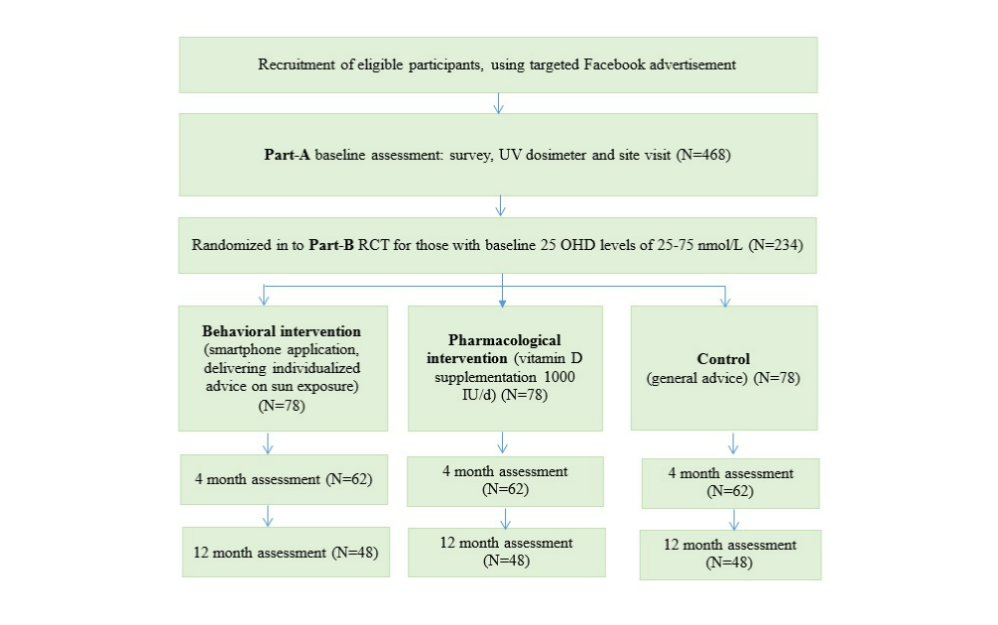
Study flowchart of participants.

In addition, site visits take place at our study center at each time point to collect a range of clinical health information, including blood tests, bone density (baseline and 12 months), and tests of muscle health (Figure 1).

### Subject Selection

Participants in this study are female, aged between 16 to 25 years upon inclusion in the study, and residing in Victoria, Australia, for the duration of the study. Inclusion criteria are completion of all components of part A of the Safe-D study, serum 25 OHD levels between 25 and 75 nmol/L, plus ownership and regular use of a smartphone with Apple or Android operating systems.

Exclusion criteria include a history of skin melanoma (or having a first-degree relative (parent or sibling) who has had a melanoma), current pregnancy, breastfeeding, an intention to conceive in the next 12 months, current supplementation with ≥800 IU vitamin D daily, an intention to move out of Australia during the course of the study, any chronic health condition or medication that may disturb vitamin D metabolism or action or cause safety concerns, any medical condition, or using any medication that increases sensitivity to sun light or UV radiation.

The inclusion and exclusion criteria were designed so that any harm to study participants is minimized as far as practically possible, while selecting as broad a sample as possible to maximize the generalizability of the findings. The exclusion and inclusion criteria also assist to reduce confounding of results. If participants meet any of the exclusion criteria, at any time in the study they are withdrawn from intervention as appropriate for good clinical care. Where possible, these participants complete all data collection including study visits for the purpose of an intention-to-treat (ITT) analysis.

### Proposed Sample Size

A meta-analysis of 16 studies found an increased serum 25 OHD concentration of approximately 1 to 2 nmol/L for each 100 IU per day of supplemental vitamin D [24]. Assuming average supplementation of 1000 IU vitamin D/day, we expect changes in serum 25 OHD concentration of approximately 10 to 20 nmol/L in the pharmacological intervention group. By using ITT analysis, a sample size of 62 per arm at 4 months (corresponding to 78 per arm at baseline) will 85% power to detect a difference of 15 nmol/L in 25 OHD levels between groups (assuming a standard deviation of 25 nmol/L and total level of significance of 0.05). This gives an 80% power to detect a 15 nmol/L difference in per protocol analysis, assuming 85% adherence to the protocol, and making the assumptions of at least 50% of participants fall in the range of 25 to 75 nmol/L at entry, 20% attrition at 4 months, and 30% total attrition at 12 months [25].

We expect to be able to include 48 participants per arm (n=56 for ITT analysis) in the 12-month per protocol analyses (accounting for attrition and noncompliance), giving us 80% power (85% in ITT) at a 5% significance level to detect a 9 to 10 nmol/L difference in change from baseline between the two intervention arms (assuming a lower standard deviation of 20 nmol/L due to seasonal matching).

Based of the above, the study team aim to recruit 468 young women into part A of the study, which should lead to approximately 234 participants in part B assuming at least 50% of participants fall in the serum 25 OHD range of 25 to 75 nmol/L, meet other eligibility criteria for part B and agree to participate.

### Recruitment

Participants who complete part A of the Safe-D study (including completion of a comprehensive questionnaire, wearing of an UV dosimeter for a period of 14 consecutive days and participation in the study site visit) and meet the eligibility criteria for Part B are invited to participate in the study. Upon receipt of the serum 25 OHD and other pathology results from the laboratory, eligible participants are contacted by a member of the study team to inform them that they are eligible to participate. A verbal consent process takes place during the telephone call. Each volunteer is given sufficient time to review the detailed participant information and consent form (PICF), and is given the opportunity to ask questions about the study. Subjects are then asked to provide written informed consent in order to participate. All participants under 18 years are assessed as a mature minor and those deemed unable to provide informed consent require the consent of their parent or guardian to participate in the study [26].

All participants are offered compensation for their time in the form of an AU$30 gift voucher at each assessment. Participants identified as having high depressive and/or anxiety symptoms in study survey responses at any time point are sent information booklets (beyondblue booklet; “What works for anxiety disorders?” [27] and “What works for depression in young people”) [28]. Participants who answer positively to suicidal ideation are contacted by a study team member with Applied Suicide Intervention Skills Training, to ensure the participant’s safety and well-being [29].

Participants whose serum 25 OHD levels show moderate to severe deficiency (<25 nmol/L) are contacted by the study team and strongly advised to review the results with their primary care physician. This group is expected to be <8% of the target population and are not randomized into part B. Subjects whose levels drop below 25 nmol/L at 4 months are also immediately referred for appropriate treatment; however, they are encouraged to remain in the trial to reduce attrition.

### Verbal, Written, and Electronic Consent

Verbal consent is obtained from all participants in this study by telephone communication. Participants then are sent email links, so that they can complete the Web-based surveys. Each of the surveys includes the PICF and this is being used as a means of obtaining electronic consent.

Prior to the site visit, all participants are sent a hard copy of the PICF with a welcome letter. The PICF is reviewed at the commencement of the study visit prior to the collection of any biological data.

An electronic consent (econsent) form is offered to participants, which is obtained through a secure link sent by the study team via LimeSurvey. Information contained in the PICF is displayed on screen and requires participants to click “I agree” to a statement that they have read and understood the information and freely agree to participate in the research project. Participants giving electronic consent are asked to provide written consent at the first subsequent opportunity to do so. The econsent form allows recruitment and allocates the participants to the intervention groups to allow for a more streamlined process. Eligible participants were randomized into one of three groups.

### Trial Interventions

#### Behavioral Intervention Group

All participants randomized to the behavioral intervention group receive instructions on how to download the Safe-D study mobile-based app (Safe-D app) to use for 12 months following randomization [20]. The Safe-D app is designed for both Apple and Android operating systems.

The Safe-D app delivers advice about how to obtain safe and effective sun exposure daily, as per guidelines developed by the Safe-D team in conjunction with the SunSmart guidelines [20]. An algorithm was incorporated into the app to estimate the time required with direct exposure to sunlight to achieve adequate vitamin D levels [30]. The mechanics of algorithm are not apparent to users. It is the messages themselves that convey the complex information simply, and the use of game elements to further simplify understanding. In Safe-D app UV-exposure is shown as different shape of sunflower to users to convey message simply and effectively. Advice is tailored according to the individual’s characteristics and reported behaviors, including Fitzpatrick skin type [31], clothing, sunscreen use, and local UV forecast (location determined using the smartphone’s global positioning system or by manually entering location details) sourced from the Australia Bureau of Meteorology and the Australian Radiation Protection and Nuclear Safety Agency. As there is a need for improved education in the community about SunSmart behaviors and safe methods to achieve the best possible vitamin D production, general advice is also delivered.

The Safe-D app enables tracking of UV-exposure, records missed exposure, and monitors participant progress regarding time spent in the sun. Participants start the app timer when they are in direct sunlight and stop when they are no longer exposed. The app sends tailored messages to participants depending on their sun exposure records. The messages are sent as push notification, rather than requiring participants to check the app daily [32-34]. Educational messages are also sent to maintain participant motivation and interest in the study. Messages encourage appropriate and safe levels of UV-exposure and explain the importance of vitamin D, especially in women, health consequences of vitamin D deficiency, and tips to improve vitamin D status. Three types of messages are sent to participants through the app: automatic push notifications, automatic in-app messages, and tailored and personalized mail messages [35]. Participants are able to turn off the automatic push notification, though they are encouraged by the study team during their randomization call not to do so. Safety is monitored within the app and any participant who exceeds the recommended time in the sun receives an autogenerated safety warning and the application reports any consequences of overexposure to study staff. Use of the application is also measured by the number of times participants open it [20].

#### Pharmacological Intervention Group

All participants in this group receive a 1-year supply of 1000 IU vitamin D supplements. Participants are informed of the prescribed dose and route of administration of the supplements (oral), and the recommended storage conditions. Participants are sent weekly regular SMS text messaging with (short message service, SMS) reminders to take the equivalent dose of 1 capsule per day, and reminded that if they miss any doses throughout the week, that they may take the missed tablets all at once that day, without any risk of toxicity. Messages are sent weekly for the first month of participation, taper to fortnight, then monthly. Standard protocols for the receipt, dispensing, return, and disposal of these supplements have been developed with the assistance of a research pharmacist.

#### Control group

All participants in this group receive general advice in the form of the “How much sun is enough?” pamphlet produced by Cancer Council Victoria [36]. This pamphlet is sent to participants via email. It provides information about achieving adequate vitamin D status using safe levels of UV exposure and diet, and a contact number to obtain further advice, with links to SunSmart Victoria’s Web-based vitamin D resources.

### Randomization

Participants are randomized into one of the three intervention groups using stratified block randomization with computer-generated varying block sizes (3, 6, and 9), based on baseline serum 25 OHD levels 25 to 49 nmol/L and 50 to 74 nmol/L. The study statistician (AG) is responsible for the generation of the randomization schedule and preparing the codes. An electronic process is used to generate the codes. As each eligible participant is identified from part A of the Safe-D study and consented, the statistician is emailed by an unblinded researcher, and a randomly generated allocation group is assigned, details of which are kept in the participant’s notes and entered into the unblinded database. All other study team members who collect outcome data are blinded to intervention group allocation.

### Blinding

As far as practically possible, procedures are in place to maintain blinding of team members who collect outcome data. They are blinded to the participant’s treatment allocated Two databases are used to avoid inadvertent unblinding, one for blinded members and one for unblinded members. Participants are blinded to their vitamin D stratification, and only receive the details of their vitamin D results during the trial if clinically necessary. All vitamin D results will be provided to participants upon conclusion of the study.

### Data Collection

A wide-range of information is collected from participants throughout the course of this study [9]. These data are sourced from self-administered Web-based surveys and clinical data are obtained through site visits. Data are collected from all participants at baseline (0 month) and at the end of the study (after 12 months of study participation). All data except the bone density scans are collected at 4 months, which is the approximate duration predicted for vitamin D levels to reach a steady state with intervention [37]. A modified version of the questionnaires is used at 4 months. Bone density testing is not performed at 4 months because significant changes are unlikely to be detected at that time-point.

### Questionnaires Content

The Cancer Council Victoria Questionnaire was comprised of dietary intake and portion sizes data. The other questionnaires comprise four modules, which the participants are able to complete either altogether or on separate occasions over a 2-week period before the site visit. The modules cover groups of health areas as described as follows:

1. Module A: demography, medical history, use of health care professionals, use of medications and allergy data.
2. Module B: nutrition, dietary behaviors, and weight management data.
3. Module C: body image, alcohol use, tobacco use, and illicit drug use information.
4. Module D: diet, physical activity, pain and injuries, sun exposure, and mental health data.

### Site Visit Assessment and Rationale

Participants are asked to attend Royal Melbourne Hospital for a 2-hour study site visit baseline and 12 months for a health check including a physical examination, blood collection, silicone skin cast of the hand, skin reflectance, bone density and body composition scans, and Leonardo mechanography testing [38]. They are also asked to attend for a 1-hour visit at the 4-month follow-up for the above tests except that bone density tests are not performed at this time point.

### Physical Examination

Blood pressure, resting heart rate, waist circumference, hip circumference, height, and weight are measured for general health assessment. Blood pressure is measured twice with two different machines and recorded as systolic and diastolic blood pressure. Height is measured to the closest of 0.1 cm by using a wall-mounted stadiometer. Weight is measured to the closest of 0.01 kg.

### Blood Collection

A fasting, morning blood sample is collected to test for analytes by standard methods including 25 OHD, thyroid stimulating hormone, prolactin, insulin, HbA1c, glucose, lipids (total cholesterol, high-density lipoprotein, low-density lipoprotein, and triglyceride), calcium, parathyroid hormone, albumin, creatinine and C-reactive protein [9]. VivoPharm Laboratories employ a highly-sensitive, accurate, and precise liquid chromatography-tandem mass spectrometry (LC-MS/MS) method using Applied Biosystems 4000 Q Trap and Agilent LC-MS/MS instruments, which is used to measure serum 25 OHD3 and serum 25 OHD2 concentrations for this study. Serum 25 OHD concentration is the best indicator of vitamin D status. Ligand-binding assays are the standard method for measuring serum 25 OHD but have some limitations including poor agreement between assays and laboratories [39], inability to distinguish between serum 25 OHD2 and 25 OHD3, and systematic under- or overestimation of 25 OHD levels [40]. The current “gold standard” method for determining vitamin D levels is LC-MS/MS, which is more accurate and precise, uses standards of defined concentrations, needs smaller sample volume and shorter turnaround time than alternative methods, and distinguishes between D2 and D3 metabolites. For these reasons, we chose the LC-MS/MS method to measure vitamin D levels.

### Skin Cast of Hand

Actinic skin damage is measured at baseline, after 4 and 12 months of intervention by taking a silicone rubber cast of the dorsum of the hand to assess skin damage [41]. The Beagley and Gibson grading system is used to score the skin casts [42]. This visual system of grading is not time-consuming and it is well suited for use in studies with large sample size. Skin casts are graded on a scale of 1 to 6, with 1 indicating undamaged skin that is evenly spaced with fine lines of equal depth. Grade 6 is indicative of maximum photo-damage, specifically with a more flattened appearance to the skin surface [43]. 

### Skin Reflectance

Cutaneous melanin density is measured at each visit using a spectrophotometer as skin color is a covariate to be controlled for in assessing vitamin D response. Melanin density is measured at both a UV-unexposed region (inner side of upper arm) and exposed regions (back of hand and facial cheek).

### Bone Density and Body Composition Scans

Participants attend the Bone Densitometry Unit at the Royal Melbourne Hospital for dual energy x-ray absorptiometry (DXA) and peripheral quantitative computed tomography (pQCT) scanning. DXA is used to measure areal bone mineral density and bone mineral content as well as soft tissue composition. The parts of the body to be scanned are lumbar spine, total hip, femoral neck, and total body [44]. Peripheral QCT of the tibia is used to assess volumetric bone density, bone geometry, and muscle cross-sectional area to investigate the relationship between 25 OHD and these measures [45].

### Leonardo Mechanography

Muscle measurements are taken using a Leonardo jumping mechanography ground reaction force platform for muscle strength and muscle performance, to examine the relationship between these measurements and 25 OHD [38]. Single two leg jump, multiple one leg hop, and balance testing are performed to estimate muscle strength, efficiency of movement, maximum voluntary force, maximum acceleration, stiffness, energy storage capacity, and Esslinger Fitness Index.

### Sun Exposure/SunSmart Behavior

Real-time UVB exposure is measured objectively in all participants at baseline, at 4 and after 12 months of intervention using a small, discreet, wearable UV dosimeter, with a sampling interval of 30 seconds to provide real-time profiles of UV-exposure during 14 consecutive days before the visit. The dosimeter is worn on the wrist like a watch. Participants also complete a log and standard questionnaire about sunlight exposure and SunSmart behavior [46], for comparison with the objective data. Logs are to report clothing worn, sunscreen use, sunburn, and when they took the watch off/put on.

### Statistical Analysis

The primary outcome in this study is the change in serum 25 OHD concentration at 4 months. Secondary outcomes include: objectively measured sunlight exposure, SunSmart behavior, compliance rates for the two interventions, and musculoskeletal health measures at 12 months. Exploratory outcomes include metabolic profiles, body composition and weight, atopic/allergic symptoms, mood and mental health, knowledge about sun-safe behavior, as well as defining the determinants of vitamin D status in young women using baseline data and investigating the effects of vitamin D improvement on metabolic profiles, body composition and weight, atopic/allergic symptoms, mood, and mental health after 4 months. Main analyses will be conducted on an ITT basis to compare the intervention arms against the control group at 4 months, using various imputation strategies to account for missing data arising from sample attrition.

Due to possible protocol violations (eg, noncompliance with the prescribed treatment and unblinding occurrences), a secondary per protocol analysis designed to adjust for noncompliance will be undertaken at 4 and 12 months and results compared with the ITT analysis. Subjects in the control and behavioral intervention arms who start taking vitamin D supplements during the trial and noncompliers with the interventions will be excluded from the per protocol analysis.

Kolmogrov-Smirnov test and histogram chart will be used to assess the normality of continuous variables. Baseline general characteristics will be examined using one-way analysis of variance (ANOVA) for continuous variables and chi-square for categorical variables. Two-way ANOVA will be used to determine the effects of supplementation and behavioral intervention. Tukey's post-hoc comparisons will be used to identify pair wise differences when we reach a significant finding in multivariate regression. *P* values <0.05 will be considered as significant. All statistical analyses will be performed using the Statistical Package for Social Science version 22.

### Compliance and Withdrawal

The measurement of study compliance may vary between the three groups. The app contains built-in timers and data that allow the study team to determine how often the participants in the behavioral intervention group access the app.

Unblinded study staff undertake a manual count of the supplements at the 4 and 12 months visits, on return of the container of supplements. These data are recorded and a compliance percentage is determined. Unblinded study staff call participants in the control group to ask if they have read and understood the information brochure 2 weeks after randomization and then each month.

Any participants who have commenced vitamin D supplementation throughout the course of the study, and are not in the pharmacological intervention arm, remain in the study, and their data are analysed using the ITT analysis. Subsequently, per protocol analyses are performed, excluding the results of these participants.

Any participant who fails to meet the eligibility criteria for the duration of the study is invited to complete their remaining study visits for ITT analyses. In addition, any participant who commences participation in the study but, at any stage of the study, withdraws their consent, is withdrawn from the study. Any data that they have contributed to the study continues to be used, unless the participant states specifically that they would like their data to be deleted. This process is formally stated to the participant before they commence the study.

The study team make reasonable attempts to contact a participant before withdrawing them from the study. This includes sending emails, text messages, making telephone calls, and sending written letters to the participant’s home address. If no contact can be made with the participant using all mentioned methods three times, the participant is withdrawn from the study and a letter is sent to them to explain this decision.

### Ethical and Legal Considerations

This study has received approval from the Melbourne Health Human Research and Ethics Committee (HREC) and is conducted according to the principles and rules laid down in the Declaration of Helsinki and its subsequent amendments. It is carried out according to the revised National Statement on Ethical Conduct in Research Involving Humans (2007) produced by the National Health and Medical Research Council of Australia [38]. This national statement was developed to protect the interests of people who participate in research studies.

Mandatory reporting requirements pertaining to physical or sexual abuse of minors are adhered to by the study team and incorporated into the PICF so that participants are aware of the obligations of the study team. Data are kept confidential except if required by law. Information regarding illegal drug use may be disclosed to relevant authorities if required by law.

### Clinically-Significant Results

All participants who have an abnormal pathology result, which is considered to be clinically significant after review by the principal investigator are contacted and/or receive the results by mail or telephone, depending on the urgency of the matter. Specifically, any participant who records a serum 25 OHD result lower than 25 nmol/L is withdrawn and is referred to their treating general practitioner (GP) for advice and follow-up.

### Adverse Events

An adverse event is defined as any occurrence that has unfavorable and/or unintended effects on research subjects, regardless of severity or study-relatedness. Adverse events may manifest as new findings (signs, symptoms, diagnoses, laboratory results) or alterations in pre-existing conditions. All adverse events occurring during the study are recorded whether or not they are considered to be serious and/or related to the study. Any major adverse events are reported in writing to the Melbourne Heath HREC. Based on the self-reported and UV dosimeter data, any subjects receiving UV-B exposure at levels deemed to place them at risk are advised that they are at high risk and provided with further information about SunSmart behaviour and safe sun exposure.

## Results

Recruitment is currently underway. Publication of trial results is anticipated in 2017.

## Discussion

### Trial Implications

Causes of vitamin D deficiency include decreased sun exposure, use of UV-B blocking sunscreens, low dietary intake of vitamin D, obesity, and possibly smoking. Sunlight exposure is the major source of vitamin D (through the internal synthesis of vitamin D3), accounting for 80% to 90% of circulating vitamin D metabolites, few foods other than fatty fish contain vitamin D [47].

To the investigators’ knowledge, this study is the first randomized trial to assess the effectiveness of an eHealth lifestyle intervention to safely improve vitamin D status in young women, and addresses the limitations of previous studies that have been small, limited by the imprecision of traditional assays for measuring 25 OHD levels, and/or lacking data on adolescents and younger women. In addition, recruitment via Facebook and subsequent enrolment into an intervention trial is novel in this study. Recruitment via Facebook can be effective at reaching a large number of potential participants, as well as improving affiliated costs. Also it is affordable and reach a wide diverse population, thus increasing generalizability [48]. If successful, the smartphone eHealth intervention developed for this study would be readily deployable throughout Australia and internationally, translatable to different demographics, and has the potential to be incorporated into health promotion initiatives and clinical care. The extensive data collection being undertaken by this study allows for the identification of possible relationships between vitamin D status and a range of other health conditions. As there are seasonal differences in 25 OHD levels, all measurements are repeated after 1 year of intervention.

## Limitations

This study has a number of limitations. First, blinding is a difficult issue to address. As far as practically possible, study staff are blinded to the group to which participants are allocated throughout the study. However, we are not able to blind participants to their intervention group allocation. Another limitation of our study is using Facebook advertising for recruitment, which can cause selection bias. However, in the pilot study on feasibility for the same age group reasonable representativeness with the general population was achieved apart from selecting those with a higher level of education [10]. This is common to most methods used for recruitment of subjects from general populations.

## Abbreviations

25 OHD: 25-hydroxyvitamin D
ANOVA: analysis of variance
ANZCTR: Australian New Zealand Clinical Trials Registry
DXA: dual energy x-ray absorptiometry
HREC: Human Research and Ethics Committee
ITT: intention to treat
LC-MS/MS: liquid chromatography-tandem mass spectrometry
NHMRC: National Health and Medical Research Council
PQCT: peripheral quantitative computed tomography
PICF: participant information and consent form
SMS: short message service
UV: ultraviolet
